# Case Report: Cortical bone loss, impaired mineralization, and reduced adiposity contributing to chronic bone pain in SFRP4-related skeletal disease with an ALPL variant

**DOI:** 10.3389/fendo.2026.1870802

**Published:** 2026-06-16

**Authors:** Fernando Lizcano, Eliana Avilés, Cristian López, Silvia Maradei-Anaya, Maria Camila Ballesteros-García, Lizeth Bustamante, Fredy Luna, Miguel O’Meara, Alex Valenzuela

**Affiliations:** 1 Center of Biomedical Investigation (CIBUS), Universidad de La Sabana, Chía, Colombia; 2 Endocrinology, Fundación CardioInfantil-Instituto de Cardiología, Bogotá, Colombia; 3 Department of Genetics, Fundación CardioInfantil-Instituto de Cardiología, Bogotá, Colombia; 4 Department of Internal Medicine, Fundación CardioInfantil-Instituto de Cardiología, Bogotá, Colombia

**Keywords:** adipose tissue, alkaline phosphatase, ALPL, bone marrow edema, Pyle disease, SFRP4, Wnt signaling

## Abstract

**Background:**

Pyle disease is a rare metaphyseal dysplasia caused by loss-of-function variants in SFRP4, leading to abnormal cortical modeling with relative preservation of trabecular bone. The mechanisms involved in severe and persistent bone pain in adult patients remain incompletely understood.

**Case presentation:**

We report an adult woman with genetically confirmed homozygous SFRP4 p.Asn53Tyr–related skeletal disease presenting with marked and persistent bone pain. Dual-energy X-ray absorptiometry showed preserved lumbar bone mineral density but markedly reduced femoral neck bone mass, consistent with predominant cortical bone loss. Bone alkaline phosphatase levels were persistently low, whereas vitamin B6 levels were within normal limits, arguing against overt classical hypophosphatasia. Additional genetics analysis identified a heterozygous ALPL p.Val474Ile variant of uncertain significance.

**Results and interpretation:**

Pelvic MRI demonstrated diffuse bone marrow edema, suggesting an active stress-related process. Whole-body DXA revealed markedly reduced total fat mass (~2nd percentile), indicating reduced adiposity. Together, these observations are consistent with a conceptual model in which cortical bone loss related to SFRP4 dysfunction, potential alterations in mineralization reserve, and reduced adiposity as a possible systemic manifestation of Wnt dysregulation may contribute to chronic bone pain.

**Conclusion:**

This case suggests a structural–metabolic framework in which cortical integrity, mineralization reserve, and systemic metabolic context may interact in the generation of bone pain. The proposed model should be considered exploratory and hypothesis-generating and warrants further genetic, functional, and clinical validation.

## 1 Introduction

Pyle disease is an exceptionally rare metaphyseal skeletal dysplasia caused by loss-of-function variants in *SFRP4* (secreted frizzled-related protein 4), a key extracellular regulator of canonical Wnt signaling. Disruption of this pathway results in abnormal cortical bone modeling, metaphyseal widening, and cortical thinning, whereas trabecular bone mass may remain relatively preserved (1–3). Although the structural skeletal phenotype has been well characterized, the mechanisms underlying severe and persistent bone pain in adult patients remain poorly understood. In particular, symptom severity may be disproportionate to the degree of radiographic deformity and areal bone mineral density, suggesting that factors beyond gross skeletal architecture contribute to pain generation (4). Potential mechanisms include altered biomechanical stress distribution, impaired microdamage repair, marrow stress, and reduced mineralization reserve, particularly when additional metabolic modifiers coexist. Pathogenic variants in ALPL, encoding tissue-nonspecific alkaline phosphatase, are associated with impaired mineralization, defective pyrophosphate handling, and increased susceptibility to insufficiency fractures and bone pain (5). The coexistence of a structural dysplasia and an additional mineralization-related genetic variant raises the possibility of increased skeletal vulnerability. Here, we report an adult patient with genetically confirmed Pyle disease due to a homozygous *SFRP4* variant, severe persistent pain, diffuse bone marrow edema on MRI, and a concomitant ALPL variant, suggesting a convergent structural–metabolic model of pain generation (6–8).

## 2 Results

### 2.1 Case presentation

**Figure 1:**
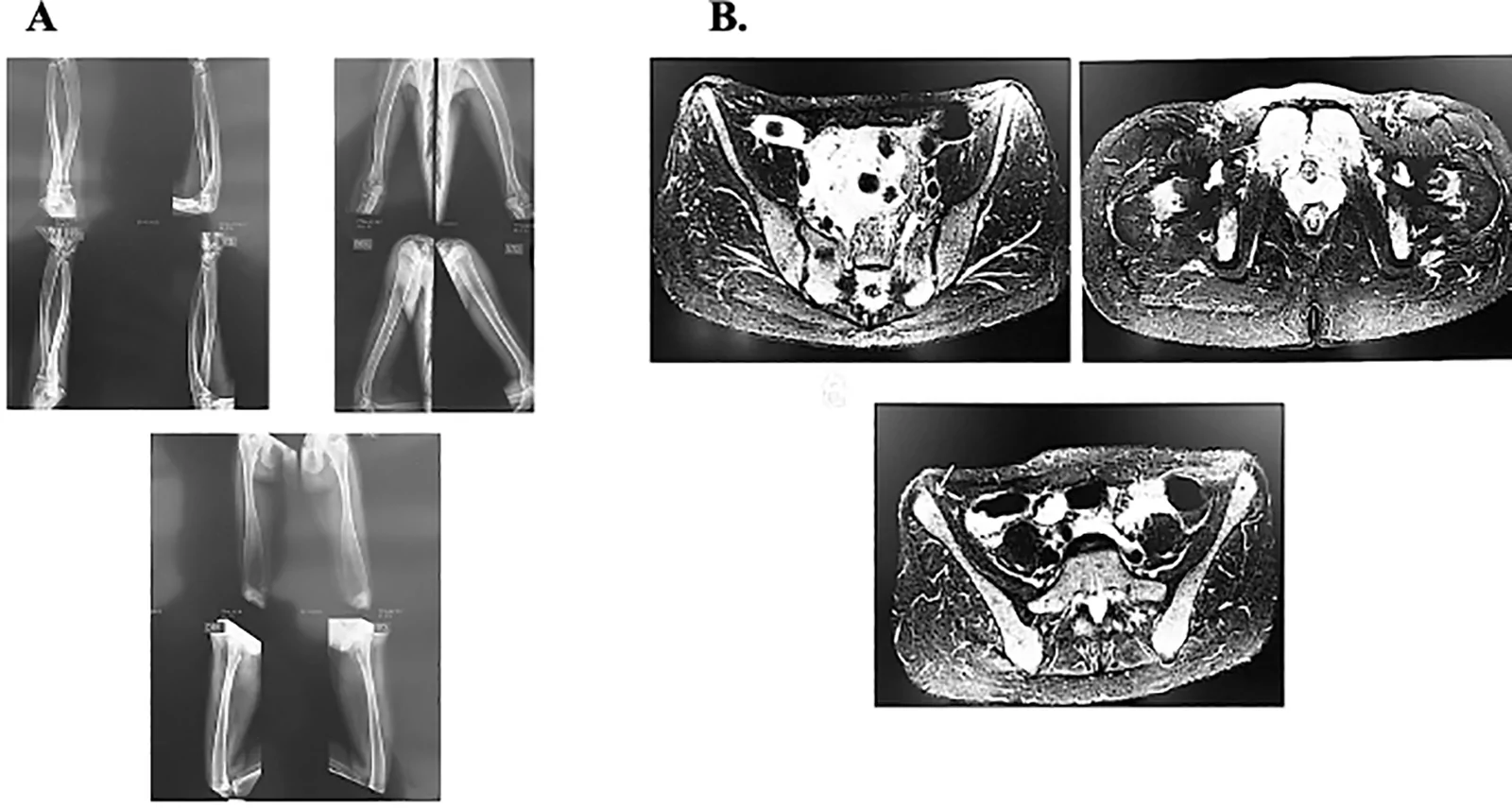
Structural and functional skeletal phenotype in the index patient with dual SFRP4 and ALPL variants. **(A)** Composite radiographic images demonstrating metaphyseal widening, cortical thinning, and Erlenmeyer flask–like deformities affecting upper and lower extremities, consistent with impaired cortical modeling in SFRP4-related Pyle disease. **(B)** Pelvic MRI (axial STIR sequences) showing diffuse bone marrow edema involving the sacrum, iliac bones, and pubic regions (arrows), without focal fracture or mass lesion, supporting an active stress-related process.

A 40-year-old woman presented with severe pain in her extremities, which made walking and normal activities difficult. The patient was initially referred for specialized evaluation because of a long-standing skeletal deformity phenotype suggestive of metaphyseal dysplasia (**Figure 1**), associated with progressive musculoskeletal pain and functional limitation. She has recently developed migraine-type headaches with anxiety and sleep disturbances. She also reports weight loss with loss of appetite and regular menstrual cycles.

An earlier genetic work-up had reportedly been interpreted as negative for a causative genetic alteration, which initially complicated diagnostic clarification and delayed etiological confirmation. Given the persistence of symptoms and the characteristic radiographic phenotype, an expanded genetic reassessment was undertaken, ultimately identifying a homozygous pathogenic *SFRP4* variant consistent with Pyle disease, together with a heterozygous *ALPL* variant. Height 1.63 m; weight 50.7 kg; BMI 19.6 kg/m²; blood pressure 130/80 mmHg; heart rate 78 bpm.

The biochemical results are presented in **Table 1**.

**Table 1:** Biochemical and hormonal evaluation of the index patient.

Parameter	Result	Reference range	Interpretation
**Total alkaline phosphatase**	**31 mU/mL**	**35–104**	**Low**
**Bone alkaline phosphatase**	**4.8 µg/dL**	**5.5–24.9**	**Low**
**Osteocalcin**	**8.1 ng/mL**	**11–43**	**Low**
Vitamin B6 (PLP)	6 ng/mL	2.1–22	Normal
Calcium	9.4 mg/dL	8.5–10.5	Normal
Phosphorus	3.3 mg/dL	2.5–4.5	Normal
PTH	43 pg/mL	15–65	Normal
25-OH Vitamin D	29.1 ng/mL	>30	Borderline low
24-h urinary calcium	33 mg/24 h	100–300	Low
24-h urinary phosphorus	289.6 mg/24 h	Variable	Within expected range
TSH	0.96 mIU/L	0.4–4.0	Normal
Estradiol	45 pg/mL	Premenopausal range	Normal
FSH	5.9 mIU/mL	Premenopausal range	Normal
LH	5.67 mIU/mL	Premenopausal range	Normal
Total cholesterol	187 mg/dL	<200	Normal
LDL cholesterol	112 mg/dL	<130	Normal
HDL cholesterol	59.9 mg/dL	>50	Normal
Triglycerides	76 mg/dL	<150	Normal
Creatinine	0.52 mg/dL	0.5–1.1	Normal

PTH, parathyroid hormone; TSH, thyroid-stimulating hormone; LDL, low-density lipoprotein; HDL, high-density lipoprotein.

Bold values indicate abnormal or clinically relevant results outside the reference range.

### 2.2 Bone marrow aspirate

#### 2.2.1 Cytological study

Trilinear representation with a maturing appearance. No morphological evidence of infiltration by hematolymphoid neoplasia.

#### 2.2.2 Flow cytometry

Myeloid precursors: 0.18%, B lymphoid precursors: CD34 + 0.06%, CD34- 0.71%; Neutrophils 64.1%, Monocytes 5.2%. Mature lymphoid population: 17.5%. Plasma cells 0.45%; Erythroid-Megakaryocyte population 10.8%. Negative result for lymphoproliferative disorder.

### 2.3 Pelvic MRI

Lumbarization of S1 is observed as an anatomical variant; diffuse alteration of bone marrow signal intensity is observed, with greater involvement of the sacrum, both iliac bones, and the pubis; less involvement of the proximal diaphysis and both femoral necks is observed, with greater representation in STIR and proton density sequences (**Figure 1**).

### 2.4 SPECT bone scintigraphy

Radiopharmaceutical: technetium-99m-labeled HMDP (99mTc-HMDP). Dose: 15.5 mCi. The physiological distribution of the radiopharmaceutical is as expected for the patient’s age. No abnormal uptake patterns suggestive of fractures were observed on the scan.

**Figure 2:**
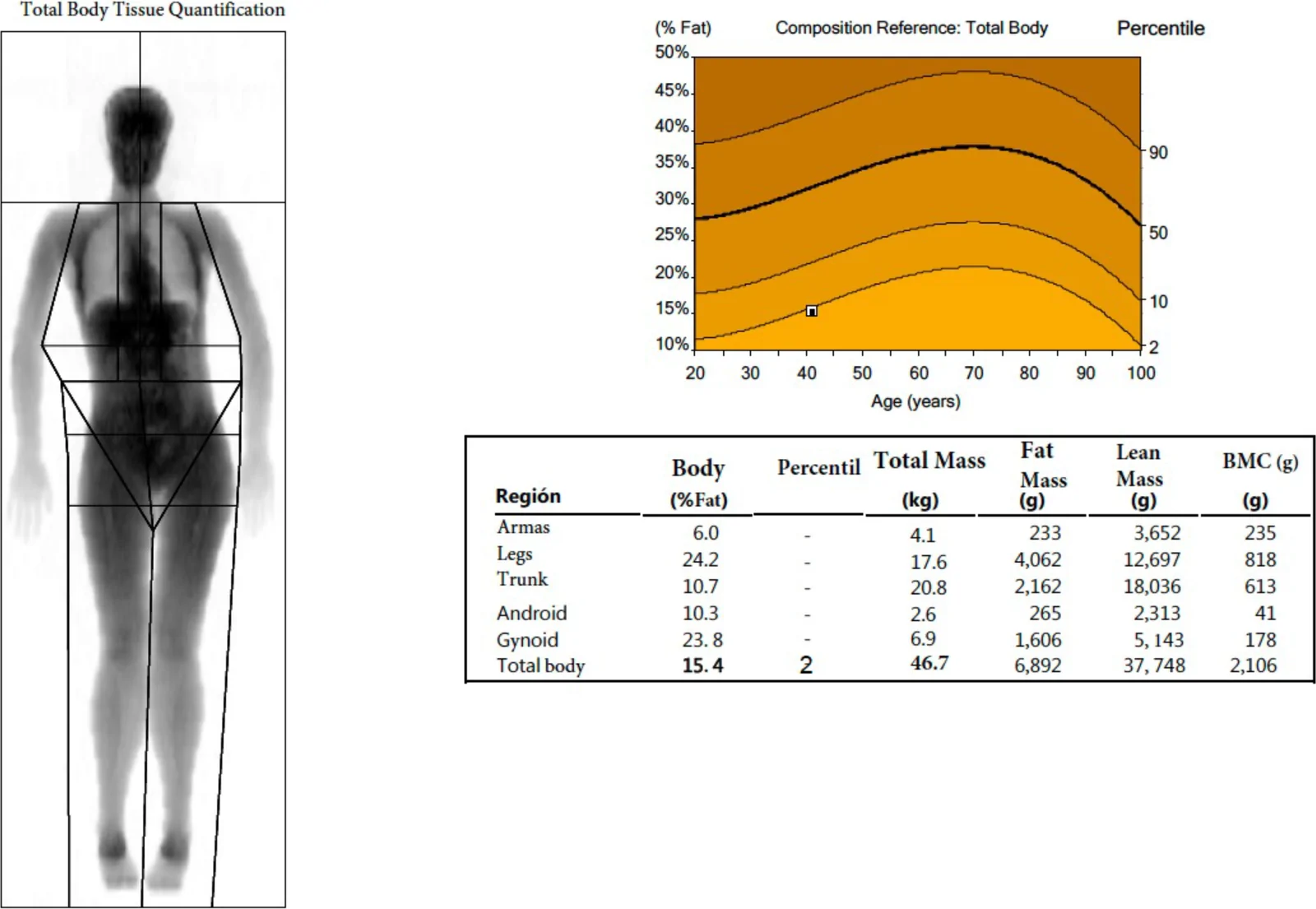
Whole-body DXA demonstrated marked reduction in total fat mass (15.4%), corresponding to approximately the 2nd percentile for age, with a fat mass index of 2.6, indicating severe depletion of adipose tissue despite preserved lean mass.

Dual-energy bone densitometry and body composition analysis were performed using a Lunar Prodigy Advance scanner. L1-L4: 1.226 g/cm², T-score 0.2; Femoral neck: 0.680 g/cm², T-score -2.6; Forearm: 0.37 g/cm², T-score -5.8. Body composition: BMI 19.6; Total weight 46.7 kg; Fat tissue 6,892 g; Lean tissue 37,748 g; bone mineral content: 2,106 g; total body fat: 15.4%, corresponding to the 2nd percentile for age. (p50 for age: 30%) Body fat mass index: 2.6 (**Figure 2**).

## 3 Discussion

The present case provides a clinically informative observation to explore potential mechanisms underlying severe pain in adult Pyle disease. While SFRP4-related dysplasia has been primarily considered a structural disorder of bone modeling, the marked discrepancy between radiographic findings and symptom severity suggests that additional factors may contribute to pain generation.

The presence of diffuse pelvic and proximal lower extremity pain, associated with functional limitation and weight loss, initially raised concern for an active bone marrow process, including hematologic infiltration or inflammatory marrow disease. However, bone marrow aspirate and flow cytometry showed no evidence of hematolymphoid neoplasia, and the absence of abnormal uptake on SPECT bone scintigraphy argued against overt fracture lines or focal osteoblastic lesions. In contrast, pelvic MRI demonstrated diffuse bone marrow edema involving the sacrum, iliac bones, pubis, and both femoral necks, with marked STIR hyperintensity, providing a direct anatomical substrate for pain generation and strongly supporting an active stress-related marrow process. This finding substantially strengthens the mechanistic interpretation of the case.

Taking these observations into account, a central mechanistic framework in this case involves the role of SFRP4 as a regulator of Wnt signaling in “cortical bone remodeling”. Loss-of-function variants in SFRP4 have been associated with abnormal metaphyseal expansion, cortical thinning, and relative preservation of trabecular bone mass. This dissociation between trabecular density and cortical integrity is highly relevant, as cortical bone is the principal determinant of resistance to bending and microfracture in weight-bearing regions such as the femoral neck (9). The markedly reduced femoral neck mineral density observed in our patient strongly supports the presence of increased cortical fragility, despite preservation of lumbar spine bone density.

From a pathophysiological perspective, we propose that defective SFRP4-mediated modulation of Wnt signaling results in abnormal cortical modeling and an increased susceptibility to repetitive microdamage formation (10). Persistent microdamage and impaired repair may contribute to activation of nociceptive pathways. However, the absence of direct inflammatory measurements in this study warrants cautious interpretation, and these mechanisms should be considered plausible but unproven.

An additional observation of interest is the presence of a heterozygous ALPL p.Val474Ile variant, currently classified as a variant of uncertain significance. Interestingly, bone alkaline phosphatase and osteocalcin levels were both reduced, a finding that may be consistent with altered osteoblast activity and reduced bone formation. Although this observation does not establish a causal relationship with the ALPL variant, it provides an additional biological context for the interpretation of the skeletal phenotype. In this context, the ALPL variant may not act as a primary driver of systemic metabolic disease, but rather as a modifier of mineralization reserve, particularly at sites exposed to chronic mechanical stress.

This concept of mineralization reserve may be especially relevant to explain pain severity. Even a partial reduction in local TNAP activity may impair hydroxyapatite deposition and delay the repair of microstructural damage, thereby perpetuating stress injury within cortical bone. We hypothesize that SFRP4-driven structural fragility and ALPL-related alterations in mineralization reserve may interact to lower the threshold for chronic microdamage persistence.

Such a mechanism may also account for the generation of pain. Persistent microdamage may activate local nociceptive and stress-related pathways. However, inflammatory mediators were not measured in this study, and therefore these mechanisms remain speculative. This framework offers a biologically plausible explanation for the disproportionate pain phenotype observed in the present case.

An additional clinically relevant finding was the marked reduction in total body fat mass, which was disproportionately low for age and body size. This observation may reflect disruption of the bone–fat axis, although causality cannot be established in a single case. Alternative explanations, including constitutional body habitus, nutritional factors, or other metabolic influences, cannot be excluded (11). Nevertheless, these findings may reflect systemic biological consequences of altered SFRP4/Wnt/β-catenin signaling beyond the skeleton (12, 13). Taken together, this case is consistent with a dual-hit threshold model, in which a primary architectural defect driven by *SFRP4* dysfunction increases biomechanical repair demand, while reduced mineralization reserve secondary to *ALPL* variation impairs effective tissue recovery, resulting in persistent bone marrow edema and clinically significant pain (14). Under physiological conditions, such microdamage is efficiently repaired; however, in this context, repair processes may be insufficient. Defective *SFRP4*-mediated regulation of canonical and non-canonical Wnt signaling likely results in abnormal cortical modeling, cortical thinning, and altered biomechanical load distribution, thereby increasing focal stress concentration in weight-bearing regions such as the sacrum and femoral neck (15–17). The concomitant ALPL variant may represent a functional modifier of mineralization reserve, potentially limiting matrix maturation and delaying repair. Although systemic biochemical markers do not support overt hypophosphatasia, subtle local effects cannot be excluded (7, 18). While circulating pyridoxal-5′-phosphate (PLP) levels remained within the normal range, arguing against overt systemic hypophosphatasia, this does not exclude a subtle reduction in tissue-nonspecific alkaline phosphatase functional reserve, particularly in a heterozygous carrier (19, 20).

Accordingly, the *ALPL* p.Val474Ile variant is best interpreted as a secondary phenotypic modifier, potentially impairing local mineralization and delaying microdamage repair under conditions of increased biomechanical stress, rather than acting as the primary disease driver (21). Within this framework, the observed bone marrow edema may represent the radiological expression of failed dynamic repair, in which structural stress exceeds the local capacity for adequate mineralized recovery (22). The genetic findings are consistent with this interpretation. The homozygous SFRP4 p.Asn53Tyr variant shows strong in silico evidence supporting its pathogenic potential and is therefore considered the most plausible genetic driver of the skeletal phenotype, whereas the ALPL variant may represent a secondary modifier influencing symptom expression (**Table 2**; **Supplementary Figure 1**). Interestingly, a historical Colombian case consistent with metaphyseal dysplasia was described in 1963. Nevertheless, the report predated molecular diagnostics and relied solely on clinical and radiographic observations; therefore, a definitive diagnosis of SFRP4-related Pyle disease cannot be established retrospectively (2).

**Table 2:** Integrated genetic and functional interpretation of SFRP4 and ALPL variants in the index patient.

Feature	SFRP4 (primary genetic driver)	ALPL (functional modifier)
Gene (HGNC)	SFRP4 (HGNC:10778)	ALPL (HGNC:438)
Transcript (MANE Select)	NM_003014.4	NM_000478.6
HGVS (cDNA; protein)	c.157A>T; p. (Asn53Tyr)	c.1420G>A; p. (Val474Ile)
dbSNP	rs1785577288	rs376354718
Zygosity	Homozygous	Heterozygous
Genomic coordinate (GRCh38)	chr7:37916381 T>A*	chr1:21577493 G>A
Population frequency (gnomAD v4.1.0)	2.05E-06 (extremely rare)	Rare
Previously reported	Not reported in ClinVar/HGMD/LOVD	Reported as VUS
CADD (PHRED)	30.0	23.3
REVEL	Deleterious	Low/tolerated
AlphaMissense (variant)	0.98 – Likely pathogenic	0.11 – Likely benign
AlphaMissense (saturation)	Multiple substitutions at Asn53 predicted likely pathogenic (0.984–0.999)	Not applicable
SIFT/PolyPhen-2	Deleterious/Probably damaging	Tolerated/Benign
In silico consensus	Strongly deleterious; highly constrained residue	Structurally benign; contextual effect plausible
ACMG criteria met	PM2, PP3, PP4; PM3/PP1 pending segregation	VUS (supporting evidence only)
Current ACMG classification	VUS → Likely pathogenic (pending segregation)	VUS
Primary biological function	Wnt signaling modulation; bone modeling	Bone mineralization (alkaline phosphatase activity)
Proposed role in this case	Primary genetic driver (Pyle disease)	Potential phenotypic modifier (hypothesis)
Phenotype correlation	Classical metaphyseal dysplasia with cortical thinning	Low bone alkaline phosphatase levels; clinical significance uncertain
Therapeutic implication	Diagnostic confirmation; genetic counseling	Supports metabolic evaluation and therapy consideration

*Genomic ref/alt alleles are reported on GRCh38. As SFRP4 lies on the negative strand, the genomic change (T>A) corresponds to the transcript-level HGVS notation c.157A>T.

**Figure 3:**
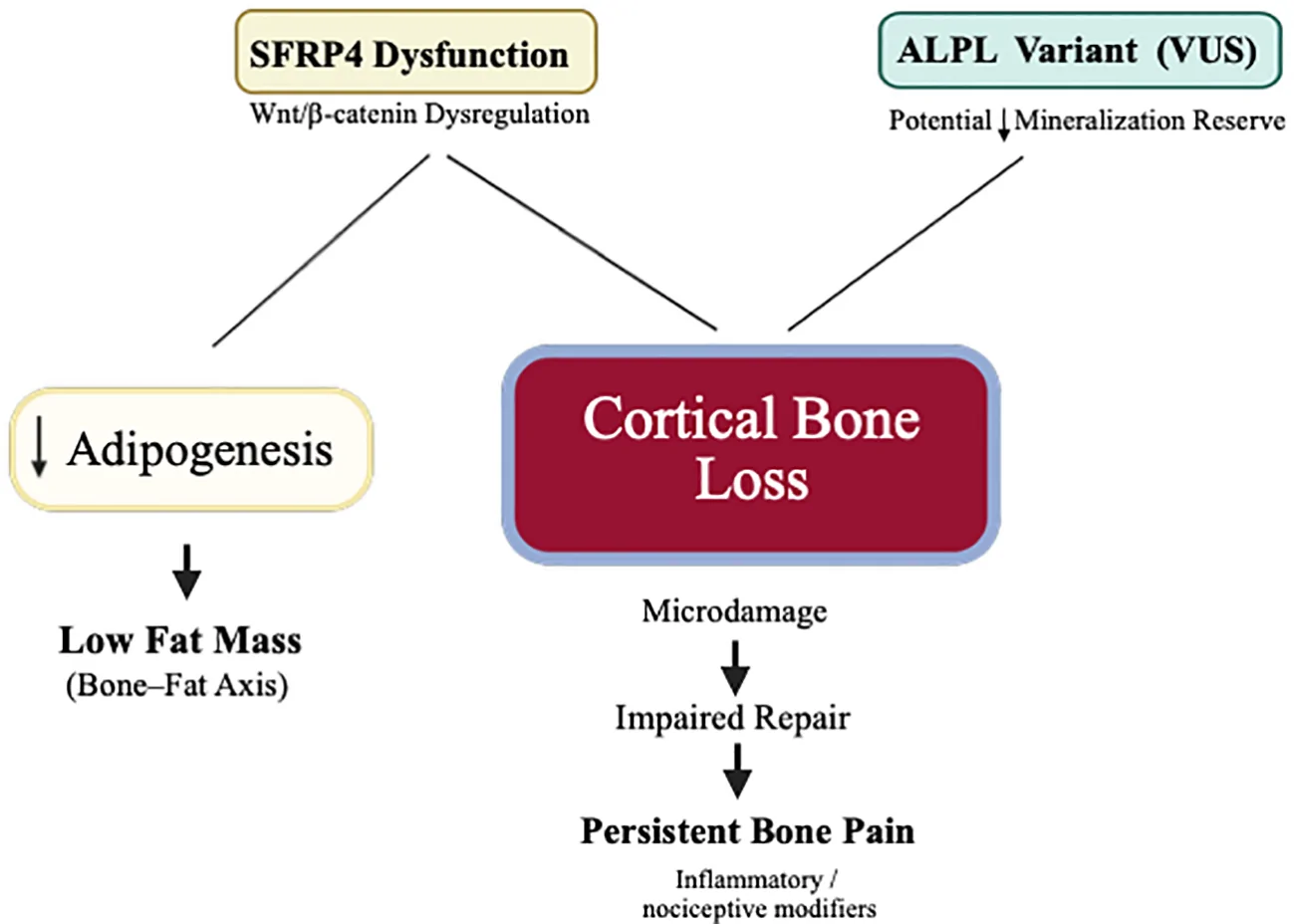
Proposed hypothesis-generating model linking SFRP4 dysfunction, cortical bone loss, reduced adiposity and chronic bone pain. These effects converge in cortical bone loss, increasing susceptibility to microdamage and impaired repair, ultimately contributing to persistent bone pain. Reduced adipogenesis and low-fat mass suggest systemic involvement of the bone–fat axis, consistent with Wnt dysregulation. Inflammatory and nociceptive modifiers may further influence pain perception.

In conclusion, this case suggests a potential structural–metabolic framework in which cortical fragility, altered mineralization reserve, and systemic metabolic factors may interact in the generation of chronic bone pain. The proposed model should be regarded as exploratory and requires genetic, functional, and clinical validation (**Figure 3**) (23).

## Data Availability

The original contributions presented in the study are included in the article and Supplementary Material. Further inquiries can be directed to the corresponding author. Because this is an individual case report, additional clinical and genetic data are not publicly available in order to protect participant privacy.

## Supplementary material

Supplementary Figure 1 In silico structural model of wild-type and mutant SFRP4 and ALPL proteins. The structural model (Alphafold3) (8) of SFRP4 (left panel) demonstrates preservation of the global secondary structure between the wild-type Asn53 and mutant Tyr53 residues, with local changes in side-chain size and interaction geometry within an α-helical region, supporting the interpretation of Asn53 as a structurally constrained residue. The ALPL structural model (right panel) shows preservation of the local secondary structure between wild-type Val474 and mutant Ile474, with subtle differences in side-chain orientation and local packing. Although no major conformational disruption is predicted, the model suggests a possible context-dependent effect on local protein stability or catalytic microenvironment, consistent with its proposed role as a functional modifier rather than a primary pathogenic driver. These in silico structural models support biological plausibility but do not constitute direct functional evidence.
