# Correction: Cortical bone loss, impaired mineralization, and reduced adiposity contributing to chronic bone pain in SFRP4-related skeletal disease with an ALPL variant

**DOI:** 10.3389/fendo.2026.1971751

**Published:** 2026-10-06

**Authors:** Fernando Lizcano, Eliana Avilés, Cristian López, Silvia Maradei-Anaya, Maria Camila Ballesteros-García, Lizeth Bustamante, Fredy Luna, Miguel O’Meara, Alex Valenzuela

**Affiliations:** 1 Center of Biomedical Investigation (CIBUS), Universidad de La Sabana, Chía, Colombia; 2 Endocrinology, Fundación CardioInfantil-Instituto de Cardiología, Bogotá, Colombia; 3 Department of Genetics, Fundación CardioInfantil-Instituto de Cardiología, Bogotá, Colombia; 4 Department of Internal Medicine, Fundación CardioInfantil-Instituto de Cardiología, Bogotá, Colombia

**Keywords:** adipose tissue, alkaline phosphatase, ALPL, bone marrow edema, Pyle disease, SFRP4, Wnt signaling

An incorrect number was provided for “Dirección de Investigación, Universidad de La Sabana”. The correct number is “MED-292-2020, project ‘Efecto de la demetilación de histonas en la diferenciación de células pancreáticas derivadas de células pluripotentes inducidas – Fase II’”. The correct **Funding** statement reads:

“The author(s) declared that financial support was received for this work and/or its publication. This work was supported by the Dirección de Investigación, Universidad de La Sabana, through grant MED-292-2020, project ‘Efecto de la demetilación de histonas en la diferenciación de células pancreáticas derivadas de células pluripotentes inducidas – Fase II.’ Additional institutional support was provided by Fundación CardioInfantil–Instituto de Cardiología and the Colombian Association of Endocrinology.”

