# Supplementary figures and images for: Case Report: Cortical bone loss, impaired mineralization, and reduced adiposity contributing to chronic bone pain in SFRP4-related skeletal disease with an ALPL variant
Source: Front Endocrinol (Lausanne). 2026 Jun 16;17:1870802. doi: 10.3389/fendo.2026.1870802 (PMC13314435; doi:10.3389/fendo.2026.1870802)

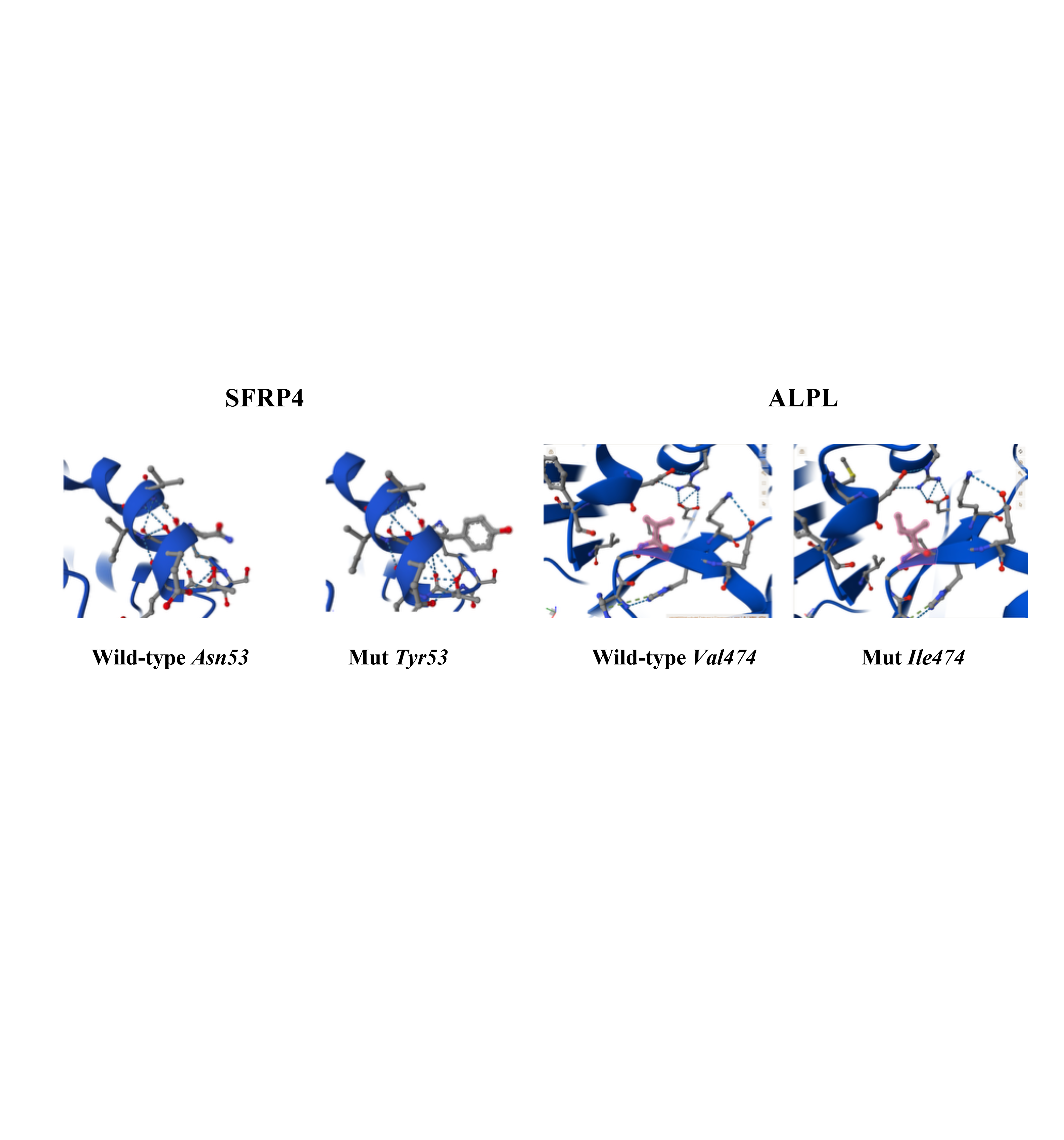

Supplement: Supplementary Figure 1 — In silico structural model of wild-type and mutant SFRP4 and ALPL proteins. The structural model (Alphafold3) (8) of SFRP4 (left panel) demonstrates preservation of the global secondary structure between the wild-type Asn53 and mutant Tyr53 residues, with local changes in side-chain size and interaction geometry within an α-helical region, supporting the interpretation of Asn53 as a structurally constrained residue. The ALPL structural model (right panel) shows preservation of the local secondary structure between wild-type Val474 and mutant Ile474, with subtle differences in side-chain orientation and local packing. Although no major conformational disruption is predicted, the model suggests a possible context-dependent effect on local protein stability or catalytic microenvironment, consistent with its proposed role as a functional modifier rather than a primary pathogenic driver. These in silico structural models support biological plausibility but do not constitute direct functional evidence. [file Image1.tiff]
